# Supplementary material for: Ramen Consumption and Gut Microbiota Diversity in Japanese Women: Cross-Sectional Data from the NEXIS Cohort Study
Source: Microorganisms. 2023 Jul 26;11(8):1892. doi: 10.3390/microorganisms11081892 (PMC10458504; doi:10.3390/microorganisms11081892)
Supplement: Supplementary file 1 [file microorganisms-11-01892-s001.zip › FIgures.pptx]

## Slide 1
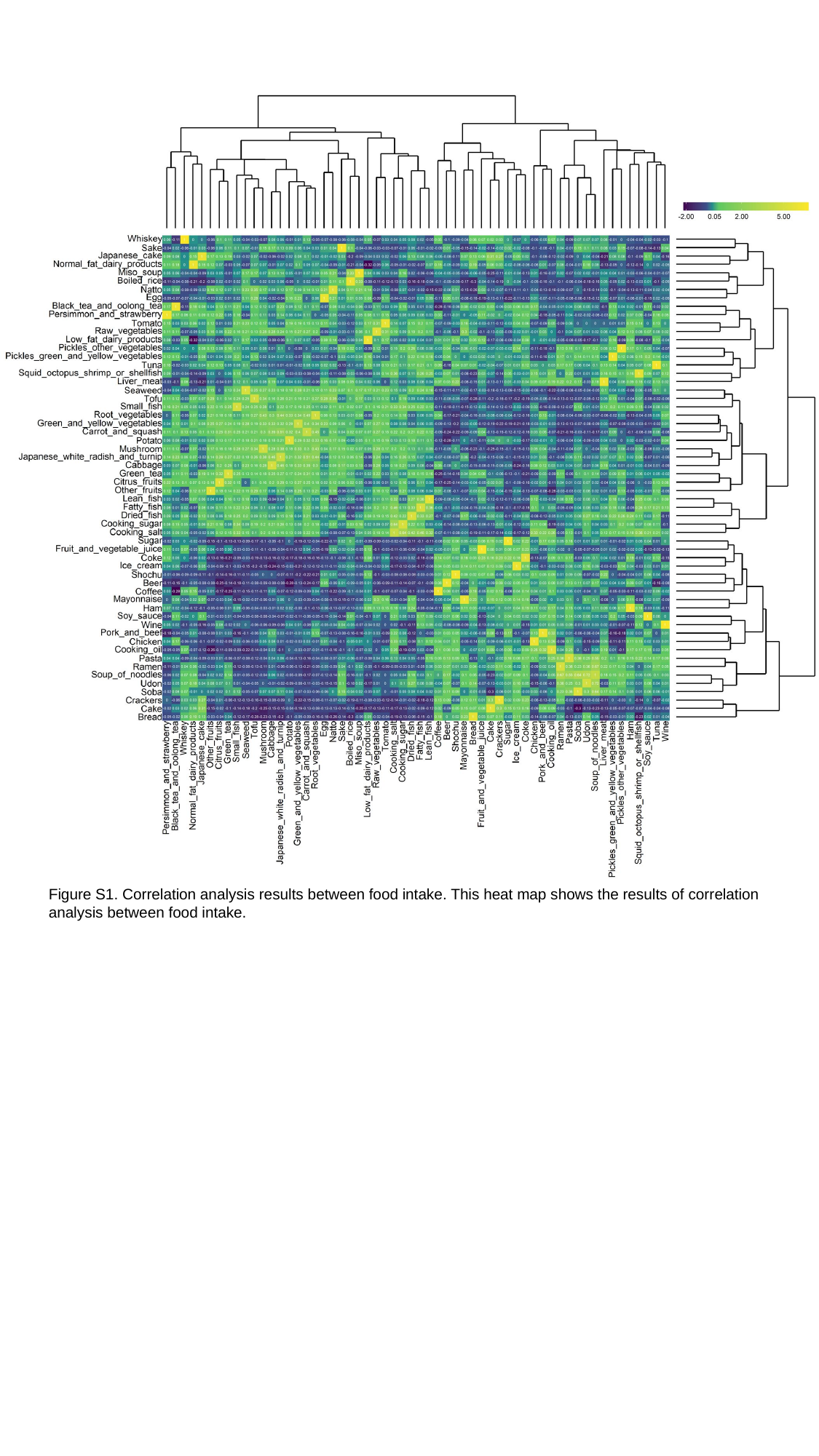

Figure S1. Correlation analysis results between food intake. This heat map shows the results of correlation analysis between food intake.

## Slide 2
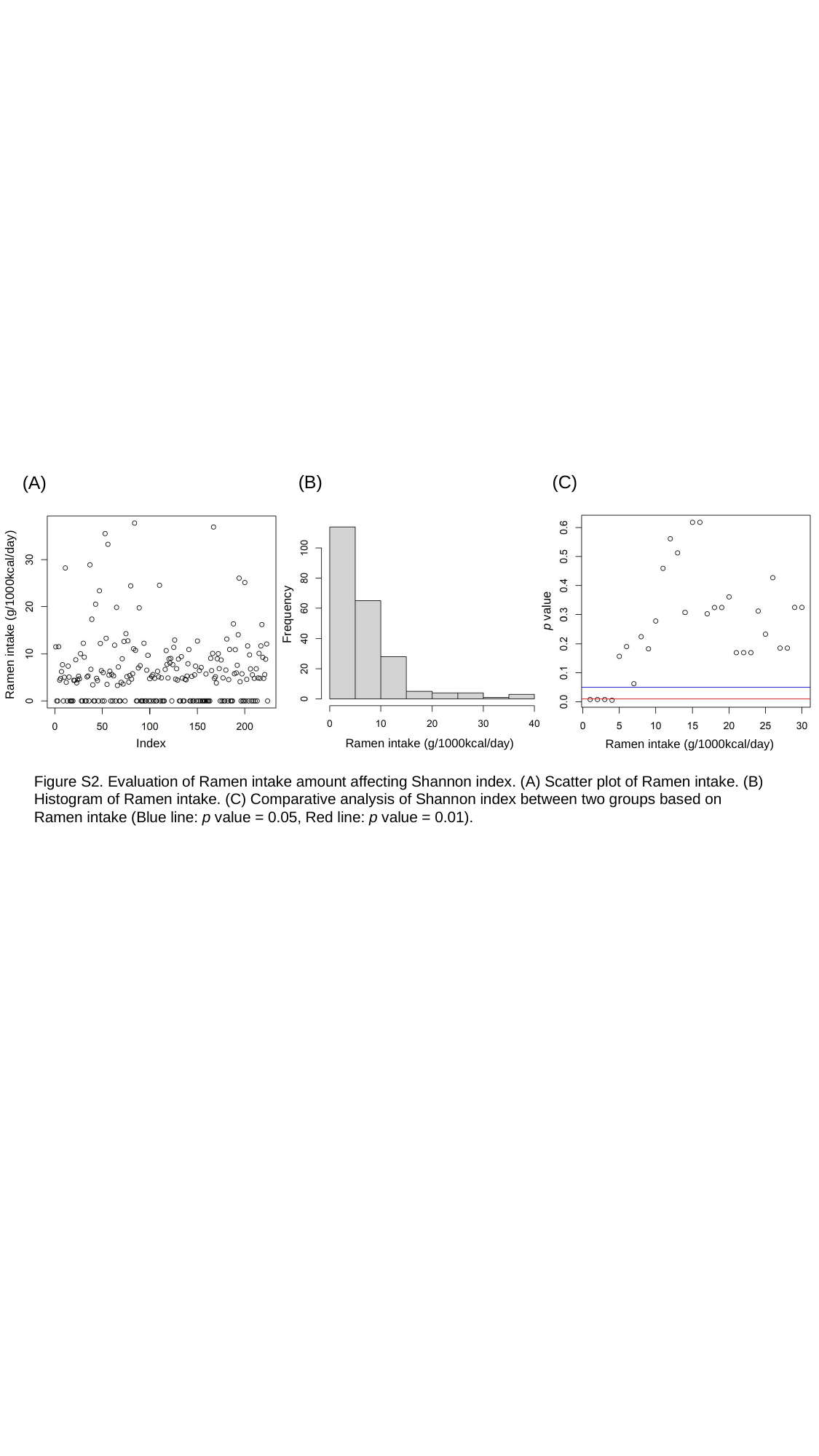

(C)
(B)
(A)
p value
Ramen intake (g/1000kcal/day)
Frequency
Index
Ramen intake (g/1000kcal/day)
Ramen intake (g/1000kcal/day)
Figure S2. Evaluation of Ramen intake amount affecting Shannon index. (A) Scatter plot of Ramen intake. (B) Histogram of Ramen intake. (C) Comparative analysis of Shannon index between two groups based on Ramen intake (Blue line: p value = 0.05, Red line: p value = 0.01).
